# Chemical analysis and biorefinery of red algae *Kappaphycus alvarezii* for efficient production of glucose from residue of carrageenan extraction process

**DOI:** 10.1186/s13068-016-0535-9

**Published:** 2016-06-10

**Authors:** Fernando Masarin, Fernando Roberto Paz Cedeno, Eddyn Gabriel Solorzano Chavez, Levi Ezequiel de Oliveira, Valéria Cress Gelli, Rubens Monti

**Affiliations:** Departamento de Bioprocessos e Biotecnologia, Faculdade de Ciências Farmacêuticas-FCF, UNESP-Univ Estadual Paulista, 14800-903 Araraquara, SP Brazil; Departamento de Alimentos e Nutrição, Faculdade de Ciências Farmacêuticas-FCF, UNESP-Univ Estadual Paulista, 14800-903 Araraquara, SP Brazil; Departamento de Engenharia Química, Escola de Engenharia de Lorena, USP-Universidade de São Paulo, CP 116, 12602-810 Lorena, SP Brazil; Instituto de Pesca-Núcleo de Pesquisa e Desenvolvimento do Litoral Norte-Agência Paulista de Pesquisa Agropecuária-Secretaria de Agricultura e Abastecimento do Estado de São Paulo, São Paulo, Brazil

**Keywords:** *Kappaphycus alvarezii*, Chemical composition, Carrageenan, Residue, Digestibility, Glucose, Bioproducts

## Abstract

**Background:**

Biorefineries serve to efficiently utilize biomass and their by-products. Algal biorefineries are designed to generate bioproducts for commercial use. Due to the high carbohydrate content of algal biomass, biorefinery to generate biofuels, such as bioethanol, is of great interest. Carrageenan is a predominant polysaccharide hydrocolloid found in red macroalgae and is widely used in food, cosmetics, and pharmaceuticals. In this study, we report the biorefinery of carrageenan derived from processing of experimental strains of the red macroalgae *Kappaphycus alvarezii*. Specifically, the chemical composition and enzymatic hydrolysis of the residue produced from carrageenan extraction were evaluated to determine the conditions for efficient generation of carbohydrate bioproducts.

**Results:**

The productivity and growth rates of *K. alvarezii* strains were assessed along with the chemical composition (total carbohydrates, ash, sulfate groups, proteins, insoluble aromatics, galacturonic acid, and lipids) of each strain. Two strains, brown and red, were selected based on their high growth rates and productivity and were treated with 6 % KOH for extraction of carrageenan. The yields of biomass from treatment with 6 % KOH solution of the brown and red strains were 89.3 and 89.5 %, respectively. The yields of carrageenan and its residue were 63.5 and 23 %, respectively, for the brown strain and 60 and 27.8 %, respectively, for the red strain. The residues from the brown and red strains were assessed to detect any potential bioproducts. The galactan, ash, protein, insoluble aromatics, and sulfate groups of the residue were reduced to comparable extents for the two strains. However, KOH treatment did not reduce the content of glucan in the residue from either strain. Glucose was produced by enzymatic hydrolysis for 72 h using both strains. The glucan conversion was 100 % for both strains, and the concentrations of glucose from the brown and red strains were 13.7 and 11.5 g L^−1^, respectively. The present results highlight the efficiency of generating a key bioproduct from carrageenan residue.

**Conclusions:**

This study demonstrates the potential for glucose production using carrageenan residue. Thus, the biorefinery of *K. alvarezii* can be exploited not only to produce carrageenan, but also to generate glucose for future use in biofuel production.

## Background

The use of algal biomass as feedstock for the food, cosmetics, nutraceutical, pharmaceutical, biofertilizer, and biofuel industries is of great interest and is an actively investigated field of research [1–4]. The concept of utilizing algae in biorefineries is a promising and economically viable alternative for the production of bioproducts, such as those crucial to biofuel production. Moreover, the cultivation of algae offers environmental appeal since growing biomass captures CO_2_, a greenhouse gas (GHG), from the atmosphere via photosynthesis. These carbon sinks can help to mitigate global warming (GW) [5–8]. Algae also produce more oxygen than consumed in respiration, unlike terrestrial plants. Furthermore, the production of cultivated algae is 22 kg m^2^ year^−1^ compared to land plants, with an average production of 0.5–4.4 kg m^2^ year^−1^ [8–10]. Thus, algal biomass is poised to provide many environmental and economic benefits.

Marine macroalgae can be classified into three major groups based on the lack or presence of phytopigments other than chlorophyll: brown algae (Phaeophyceae), green algae (Chlorophyceae), and red algae (Rhodophyceae) [11, 12]. Brown macroalgae comprises almost 1800 species with an olive-green to dark brown color derived from an abundance of fucoxanthin, a yellow–brown pigment that masks the green color of chlorophyll. The composition of brown macroalgae such as Laminaria includes up to 55 % dry weight of carbohydrates including alginate, cellulose, laminarin that can be easily hydrolyzed by laminarase (endo-1,3(4)-β-glucanase) and cellulases (endo-1,4(4)-β-glucanase) to release glucose monomers [11–13]. Green macroalgae include almost 1500 species, and are primarily composed of starch for food reserves with cellulose and pectin as the main structural polysaccharides in the cell wall [11, 12]. Red macroalgae comprise almost 6000 species of algae having a characteristic red or pink color derived from the pigments phycocyanin and phycoerythrin, which allow growth in relatively deep waters. The composition of red macroalgae varies from species to species but generally comprises cellulose, glucan, and galactan. The cell wall of red seaweed is constructed of cellulose and two kinds of long-chain structural polysaccharides that are valued for their gel-forming ability, i.e., agar and carrageenan. Agar can be readily hydrolyzed to release the galactose subunits. Carrageenan can be classified as lambda (l), kappa (k), or iota (i) based on the-gel-forming ability and is used mainly for thickening foods such as yogurt, ice cream, and pudding [11, 12].

The replacement of fossil fuels with biofuels derived from algae reduces GHG emissions from transportation [14, 15]. The high potential for production of biofuel production from algae is due to the considerable amounts of carbohydrates (found especially in macroalgae) and oil (found especially in microalgae) in these species, thus making algal biomass an excellent resource for bioethanol and biodiesel production [16–18].

Second generation biofuels based on waste biomass do not compete directly with food sources, and are thus advantageous alternatives to first generation biofuels that require large areas of farmland to dually provide food and biomass for fuel production. However, the pre-treatment phase required to convert complex carbohydrates into fermentable sugars results in low yield and high cost and serves as a technological bottleneck [19, 20]. Indeed, this challenge can be resolved using algae since the algal cell wall is virtually free of structural biopolymers such as lignin and hemicellulose (a branched carbohydrate polymer). The elimination of chemical pre-treatment steps results in minimized recalcitrance of the biomaterial and enables direct enzymatic hydrolysis of polysaccharide fractions, resulting in monomeric sugars [21].

In addition, other bioproducts can be extracted from macroalgae, such as agar, carrageenan, and alginate hydrocolloids, which are all extensively used as viscosity modifying agents in foods and cosmetic products [22]. Populations from the Scandinavian Peninsula first used carrageenan, a polysaccharide of galactose obtained from the red macroalgae *Kappaphycus alvarezii* more than five centuries ago, as a food source [22–24]. Moreover, there is recent research describing the potential of carrageenan for bioethanol production due to its high galactose content [25–30]. Biofuels produced from macroalgae are considered third generation [25–30]. However, because carrageenan is used as a food source, its use as a fuel source brings up the food versus fuel dilemma, thereby raising the challenge to find additional yet efficient uses of this valuable bioproduct.

The aim of this study is to evaluate the chemical composition of *K. alvarezii* strains and the potential digestibility of the residue generated from carrageenan processing for the production of monomeric sugar bioproducts.

## Methods

### Raw material and biomass preparation

Four different *K. alvarezii* seaweed strains were used. The strains were obtained from the Fisheries Institute, Ubatuba, São Paulo (SP). The following *K. alvarezii* strains were used: brown, red, green, and G11. The strains were grown during May and June of 2013. The *K. alvarezii* strains were grown in the Atlantic Ocean in the experimental field base at Itaguá beach in Ubatuba, SP, Brazil (GPS coordinates 23°27′5,8″S; 45°02′49,3″W). The structure used to grow the seaweed strains consisted of a raft anchored in the bay [31, 32]. Ten shoots of vegetative growth from each strain (approximately 70 g on wet basis) were pre-weighed and bound on a nylon line in a sub-assembly on the surface of the seawater, which provided a cultivation density of 6.7 plants per m^2^. For cultivation, the strains remained in the structure for 30 days. After 30 days, the strains were weighed again. The wet weight and dry mass from each strain were determined using an average humidity of 35 % (commercial value) [30, 31]. The growth rate was calculated according to the equation: ${\text{Growth}}\,{\text{rate}}\,({\text{percentage}}\,{\text{on}}\,{\text{day}} - 1) = [\left( {w_{t} /w_{0} } \right)1/t - 1]*100\,,$ where *w*_*t*_ is the final wet mass (g); *w*_*0*_ is the initial wet mass (g); and *t* is the cultivation time (30 days) [32, 33]. The productivity was calculated according to the equation: ${\text{Productivity }}({\text{gm}}^{2} \,{\text{day}}^{ - 1} ) \, (w/w,\,\,{\text{dry basis}}) = [({\text{dwtf}} - {\text{dwti}})/t*({\text{dwt}}/{\text{wwt}})]/A,$ where dwtf is the final dry mass (g); dwti is the initial dry mass (g); *t* is the cultivation time (30 days); dwt = total dry mass; wwt is the total wet mass and *A* is the total area of cultivation [32, 33]. After collection, the biomass was dried at 25 °C. The biomass was washed with distilled water, with stirring, in a 10 L polypropylene beaker for 45 min at a ratio of 35 g (dry weight) of macroalgae biomass to 1 L of distilled water. After washing, the solution was removed using a sieve with a 1 mm screen. Washing was repeated until the electrical conductivity of the wash solution was similar to that of distilled water (measured with a portable conductivity meter). After washing, the samples were again dried at 25 °C. These materials are hereafter termed ‘untreated material’.

### Chemical composition of the samples

Hexane-soluble extractives were determined by extraction with 99 % (v/v) hexane in a Soxhlet apparatus [26]. The samples were air dried, milled, and passed through a 0.84 mm screen. Approximately, 1 g of the milled sample was extracted with 99 % hexane for 8 h in the Soxhlet apparatus. The percentage of lipids was determined based on the dry weights of the extracted and non-extracted milled samples [data provided as mean ± standard deviation (SD)]. This procedure was conducted in triplicate.

The milled samples were hydrolyzed with 72 % (w/w) sulfuric acid at 30 °C for 1 h (3 mL of acid to 300 mg of sample) as described previously [34, 35]. The acid hydrolysate was diluted with 79 mL of distilled water (4 % (w/w) sulfuric acid), and the mixture was autoclaved at 121 °C for 1 h. The residual material was cooled and filtered through a porous glass filter (Scott number 3, Germany). The solids were dried to a constant weight at 105 °C and were assessed as the insoluble aromatics component. The filtrate was further passed through 0.45 µm membranes. The total sugar content in the same solution was determined by the sulfuric acid/phenol method, using sucrose as the calibration standard [36]. The filtrates were evaluated via HPLC/MS analysis (using HPLC Agilent 1200 Series and AB Sciex QTRAP mass spectrometers) to confirm the presence of monomeric sugars. Detection of the monomeric sugars in the soluble fraction was performed using HPX87P columns (Bio-Rad; Hercules, CA, USA) at 80 °C by elution with water at a rate of 0.6 mL min^−1^. The mass spectrometer was operated using electrospray ionization (ESI) in positive and negative modes. The ionization source parameters in negative mode were: ion spray: −4500 V; curtain gas: 15 psi; temperature: 650 °C; gas 1:50 psi; gas 2:50 psi; and heater interface: on. The ionization source parameters in the positive mode were as follows: ion spray: 5500 V; curtain gas: 15 psi; temperature: 650 °C; gas 1:50 psi; gas 2:50 psi; heater interface: on. The standards were diluted to 1 mg L^−1^ in water with 0.1 % acetic acid, and the optimization was performed by direct infusion into the automatic flow (10 L min^−1^) using a syringe. All sugars were detected as water adducts (+18) [M + 18]^+^ in positive mode. Xylose: (SRM1, Q1 = 168.1, Q3 = 150.0, DwellTime(ms) = 250, SD(V) = 16, EP(V) = 5, CEP(V) = 10, EC(V) = 9 and CXP(V) = 4); (SRM2, Q1 = 168.1, Q3 = 73.2, DwellTime(ms) = 250, SD(V) = 16, EP(V) = 5, CEP(V) = 10, EC(V) = 19 and CXP(V) = 4. Arabinose: (SRM1, Q1 = 168.1, Q3 = 50.1, DwellTime(ms) = 250, SD(V) = 6, EP(V) = 3.5, CEP(V) = 14, EC(V) = 9 and CXP(V) = 4), (SRM2, Q1 = 168.1, Q3 = 73.2, DwellTime(ms) = 250, SD(V) = 16, EP(V) = 5, CEP(V) = 10, EC(V) = 19 and CXP(V) = 4; (SRM2, Q1 = 168.1, Q3 = 73.0, DwellTime(ms) = 250, SD(V) = 16, EP(V) = 3.5, CEP(V) = 14 EC(V) = 21 and CXP(V) = 4 cellobiose: (SRM 1, Q1 = 360.2, Q3 = 163.2, DwellTime(ms) = 250, SD(V) = 21, EP(V) = 4.5, CEP(V) = 16, EC(V) = 17 and CXP(V) = 4), (SRM2, Q1 = 360.2, Q3 = 84.9, DwellTime(ms) = 250, SD(V) = 21, EP(V) = 4.5, CEP(V) = 16, EC(V) = 33 and CXP(V) = 4 Galactose: (SRM1, Q1 = 198.0, Q3 = 163.1, DwellTime(ms) = 250, SD(V) = 21, EP(V) = 6, CEP(V) = 12, EC(V) = 11 and CXP(V) = 4), (SRM2, Q1 = 198.0, Q3 = 91.2, DwellTime(ms) = 250, SD(V) = 21, EP(V) = 6, CEP(V) = 12, EC(V) = 19 and CXP(V) = 4 Glucose: (SRM1, Q1 = 198.1, Q3 = 85.1, DwellTime(ms) = 250, SD(V) = 16, EP(V) = 6.5, CEP(V) = 10, EC(V) = 21 and CXP(V) = 4), (SRM2, Q1 = 198.1, Q3 = 163.2, DwellTime(ms) = 250, SD(V) = 16, EP(V) = 6.5, CEP(V) = 10, EC(V) = 11 and CXP(V) = 4. Mannose: (SRM1, Q1 = 198.1, Q3 = 163.2, DwellTime(ms) = 250, SD(V) = 21, EP(V) = 8, CEP(V) = 12, EC(V) = 11 and CXP(V) = 4); (SRM2, Q1 = 198.1, Q3 = 85, DwellTime(ms) = 250, SD(V) = 21, EP(V) = 8, CEP(V) = 12, EC(V) = 25 and CXP(V) = 4. Rhamnose: (SRM1, Q1 = 198.1, Q3 = 163.2, DwellTime(ms) = 250, SD(V) = 21, EP(V) = 8, CEP(V) = 12, EC(V) = 11 and CXP(V) = 4, (SRM2, Q1 = 198.1, Q3 = 85, DwellTime(ms) = 250, SD(V) = 21, EP(V) = 8, CEP(V) = 12, EC(V) = 25 and CXP(V) = 4. Galacturonic acid was detected as [M–H]^−^ in negative mode. Galacturonic acid: (SRM1, Q1 = 193.02, Q3 = 113, DwellTime(ms) = 2500, SD(V) = −20, EP(V) = −3.5, CEP(V) = −10, EC(V) = −18 and CXP(V) = −2), (SRM2, Q1 = 193.021, Q3 = 59.1, DwellTime(ms) = 2500, SD(V) = −20, EP(V) = −3.5, CEP(V) = −10, EC(V) = −26 and CXP(V) = 0. The concentrations of monomeric sugars in the soluble fraction were determined by HPLC (HPX87P column; Bio-Rad, Hercules, CA, USA) at 80 °C using water as the eluent at a flow rate of 0.6 mL min^−1^. Sugars were detected using a temperature-controlled refractive index detector at 45 °C. Glucose, xylose, mannose, and galactose were used as external calibration standards. Corrections were performed by considering the anhydrogalactose-degradation reactions that took place during acid hydrolysis. Under the present acid hydrolysis conditions, all the anhydrogalactose present in the sample is degraded [29, 37]. Thus, the anhydrogalactose content in the carrageenans and agars was calculated using the galactose to anhydrogalactose ratio of 1:1.27 [29, 37]. The factor used to convert the sugar monomers to anhydromonomers was 0.9 for glucose and galactose. This procedure was conducted in triplicate. Glucose was reported as glucan and galactose and anhydrogalactose as galactan after correction by the hydrolysis factor. The concentration of hydroxymethylfurfural and furfural in the soluble fractions was determined using an HPLC instrument equipped with a 250 mm long column with an outer diameter of 4 mm (Hypersil; Thermo-Scientific) using acetonitrile:water (1:8) containing 1 % (v/v) acetic acid as an eluent at a flow rate of 0.8 mL min^−1^. Hydroxymethylfurfural and furfural were detected at 276 nm.

The sulfate group content of the samples was quantified using modified spectrophotometric methods [38–40]. About 0.05 g of the milled sample was weighed and placed in test flasks. One milliliter of 0.5 N HCl was added to each flask, and the flasks were capped with aluminum foil and autoclaved at 120 °C for 1 h at 1 atm. At the end of the reaction, the sample was transferred into 15 mL Falcon tubes. Water was then added to each tube to achieve a volume of 10 mL followed by centrifugation at 7000×*g* for 10 min. The supernatant (2 mL), 18 mL of distilled water, and 2 mL of 0.5 N HCl were added to the test flasks and stirred for a few seconds. Afterwards, 1 mL of BaCl_2_ gelatin (Difco-Laboratories, Detroit, EUA) was added. The tubes were kept at 25 °C for 30 min with stirring, and absorbance readings were taken at 550 nm (Genesys 10S, Thermo-Scientific).

The protein content of the samples was quantified using a Kjeldahl digester to determine the total nitrogen. The protein content was calculated using a nitrogen conversion factor of 6.25 [41]. The experiments were performed in triplicate. For quantification of the ash content of the samples [42], approximately 1 g of milled sample was weighed into a porcelain crucible and combusted in a muffle furnace at 575 ± 25 °C for 3 h using a pre-programmed heating ramp. At the end of 3 h, the pots were kept in the oven until the temperature was about 105 °C. The crucibles were cooled and weighed. The experiments were performed in duplicate. The metal content of the samples was also quantified by treating approximately 0.05 g of the milled sample with 1 mL of sulfuric acid and 2 mL of nitric acid in a glass digester at 150 °C until the solutions become clear. These solutions were allowed to swell in a 100 mL volumetric flask and analyzed using a spectrophotometer (ICP Optima Perkin Elmer Model 8000). The following metals were analyzed: manganese, calcium, sodium, copper, silicon, iron, and potassium. The experiments were performed in triplicate.

### Carrageenan processing for selected samples

Two strains of previously selected *K. alvarezii* (brown and red, grown in May 2013) were processed and the semi-refined carrageenan was extracted; the residue from this extraction process was also analyzed. Prior to extraction of the semi-refined carrageenan, “cold” alkali transformation was performed [32, 38]. Briefly, approximately 8 g (dry weight) of macroalgae was soaked in 96 mL of 6 % KOH solution (w/v) for 24 h at 25 °C (“cold” alkali transformation). The material was copiously washed with water, sun bleached for 12 h, and dried at 60 °C until constant weight was achieved. The material was weighed, milled, and passed through a 0.84 mm screen. Approximately, 3 g (dry weight) of the material obtained after alkaline transformation was extracted with 240 mL of distilled water in flasks and incubated at 65 °C for 2 h with rotary agitation at 120 rpm. The solution was then filtered using nylon tissue, and the extract was dried at 60 °C until constant weight was achieved (hereafter referred to as semi-refined carrageenan). The material retained on the nylon tissue after filtering was recovered and dried to constant weight at 60 °C (hereafter referred to as residue). Both the semi-refined carrageenan and the residue were weighed. The yield from the alkali treatment was determined from the difference between the original (untreated material) and final weights (dry weight basis). The partial yield of the semi-refined carrageenan and residue was obtained from the difference between the initial weight [the material treated with 6 % KOH (w/v)] and final weight of semi-refined carrageenan and residue (dry weight basis). The overall yields of the semi-refined carrageenan and residue were obtained from the difference between the original weight (untreated material) and final weight of the semi-refined carrageenan and residue (dry weight basis).

### Enzymatic hydrolysis of the selected samples

Enzymatic hydrolysis was performed using commercial enzyme preparations (Cellic CTec II, Novozymes, Denmark) at a dosage of 10 FPU per gram of sample (dry weight basis), corresponding to 200 IU of β-glucosidase. The total cellulases and β-glucosidase activity determined using the Celic CTec II extract were 92 FPU mL^−1^ and 1800 UI mL^−1^, respectively. Each hydrolysis experiment was conducted in 50 mL Falcon tubes containing 200 mg of milled sample (dry weight basis) and 10 mL of 50 mM sodium-acetate buffer (pH 4.8) in addition to the enzyme solution. The flasks were incubated at 45 °C with rotary agitation at 120 rpm. The reaction was stopped at defined periods from 4 to 72 h by heating the flask to 100 °C for 5 min, followed by centrifugation of the material at 7000×*g* for 10 min. The soluble fractions were assayed for glucose using HPLC with an HPX87P column (Bio-Rad) at 45 °C using water as an eluent at an elution rate of 0.6 mL min^−1^. The sugars were detected using a temperature-controlled infrared detector set at 45 °C. The glucan conversion level reported herein refers to the conversion of the polysaccharides to their monomers. Values (mean ± SD) for the hydrolysis of the samples were estimated from triplicate runs.

## Results and discussion

### Productivity and chemical composition of different *K. alvarezii* strains

**Fig. 1:**
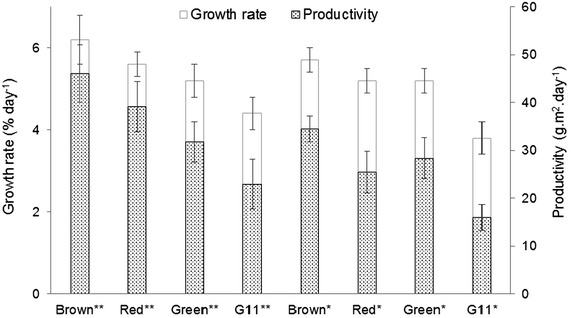
Growth rate and productivity of different strains from *K. alvarezii*. Contents present in percentage day^−1^ and g m^2^ day^−1^, respectively. *Asterisks* Cultivation for June 2013, *Two* *asterisks* cultivation for May de 2013. All reported data are the average values followed by their standard deviations

The growth rate and productivity of different *K. alvarezii* strains were evaluated based on an experimental field test in Ubatuba, São Paulo (SP), Brazil (Fig. 1). The productivity ranged from 15.9 to 46.0 g m^2^ day^−1^, and the growth rate ranged from 3.8 to 6.2 % day^−1^ (Fig. 1). The G11 strain showed the lowest productivity and growth rate of the evaluated strains. The brown and red strains grown in May 2013 showed higher productivity than the brown and red strains grown in June 2013. The average data presented were similar to those reported in the literature and the values were characteristic of *K. alvarezii* crops in the Ubatuba-SP region [32, 38].

The chemical composition of the brown and red strains grown in May 2013 and June 2013 was evaluated (Table 1). The chemical compositions of the samples varied; the samples are ranked in terms of highest productivity in Table 1. The percentages of total carbohydrate, ash, sulfate groups, proteins, insoluble aromatics, galacturonic acid, hydroxymethylfurfural, and the lipid content of the samples ranged from 51.6–55.8, 14.6–17.2, 9.6–10.8, 2.3–3.8, 1.5–3.4, 0.9–1.5, 2.8–4.5, and 0.2–1.3 %, respectively. The summative data were in the range of 88.7–95.9 %. Note that the soluble aromatics were not quantified because the UV spectrum of protein, derived from amino acids, overlaps with that of the soluble aromatics. Compounds that absorb in the visible region were also detected (data not shown), but these compounds were not quantified due to lack of a quantified, known standard. However, these compounds might account for part of the ‘undetermined compound’ content.

**Table 1:** Components of chemical composition of different strains from *K. alvarezii*

Strains	Total carbohydrates (%)	Ashes (%)	Sulfate groups (%)	Proteins (%)	Insoluble aromatics (%)	AG (%)	HMF (%)	Lipids (%)	Sum (%)
Brown^**^	53.4 ± 1.2^a,b,c,e,f^	14.6 ± 0.1^a^	9.6 ± 0.2^a,b,c,d,e,f^	3.8 ± 0.5	2.4 ± 0.1	0.9 ± 0.1	4.5 ± 0.3	0.5 ± 0.1	91.6
Red^**^	52.3 ± 1.0^b,e,f^	16.0 ± 0.1^b,c,d,e^	10.1 ± 0.3^b,c,d,e,f^	2.5 ± 0.3	1.5 ± 0.2	1.0 ± 0.1	4.1 ± 0.2	0.6 ± 0.1	88.7
Brown^*^	54.6 ± 0.4^c,d,e^	16.5 ± 0.1^c,d,e^	9.6 ± 0.8^c,d,e,f^	2.5 ± 0.5	3.0 ± 0.2	1.2 ± 0.1	3.6 ± 0.4	0.5 ± 0.1	91.5
Green^*^	55.8 ± 0.4^d^	16.4 ± 0.2^d,e^	10.7 ± 0.1^d,e,f^	3.1 ± 0.3	2.9 ± 0.2	1.5 ± 0.1	3.4 ± 0.2	1.3 ± 0.1	95.9
Red^*^	52.7 ± 1.0^e,f^	16.6 ± 0.2^e^	10.1 ± 0.1^e,f^	2.5 ± 0.3	3.4 ± 0.1	1.3 ± 0.1	2.8 ± 0.3	0.6 ± 0.1	90.0
G11^*^	51.6 ± 0.3^f^	17.2 ± 0.1^f^	10.8 ± 0.8^f^	2.3 ± 0.3	2.7 ± 0.2	1.3 ± 0.2	4.3 ± 0.1	0.2 ± 0.1	90.4

Contents present in percentage (g/100 g of original material in dry basis)

(*) Cultivation for June 2013, (**) cultivation for May 2013

*AG* galacturonic acid, *HMF* hydroximethylfurfural

All reported data are the average values followed by their standard deviations. In each column, the values with the same superscript letters do not differ among themselves at significance level of 0.05 (Tukey test, Software GraphPad Instat)

Carbohydrate constituted the main component for all strains studied. The average total carbohydrate content determined herein was 53.4 %, whereas that documented in the literature for the same species is around 63 % [26, 43]. The reported total carbohydrate content of the species *Gelidium amansii* (red macroalgae) is around 78 % [10, 29]. The highest total carbohydrate values (54.6 and 55.8 %, respectively) were obtained for the brown and green strains, both grown in June 2013 (Table 1). Ash accounted for the second major component of the samples (Table 1). The brown strain grown in May 2013 had the lowest ash content (14.6 %) and the G11 strain grown in June 2013 had the highest ash content (17.2 %). The ash contents of the other strains of the same species did not differ significantly. On average, the observed ash contents were similar to that reported in the literature for the same species [26, 43]. The species *Gelidium amansii* (red macroalgae) was recently reported to have an ash content of around 6 % [10, 29]. Notably, after harvesting algal biomass from the sea, washing with water at 25 °C is required to remove excess salts that accumulate in the biomass. The average ash content of the different strains evaluated in this study before washing was 50 %. Sulfate groups constituted the third largest component (Table 1). The average content of sulfate groups in carrageenan originating from *K. alvarezii* was 10.1 %, and there was no significant difference among the strains. The other components assessed were proteins, insoluble aromatics, galacturonic acid, hydroxymethylfurfural, and lipids (Table 1). The average protein content was 2.8 %, while that documented in the literature for the same species was around 4.5 % [26, 43]. The brown strain cultivated in May 2013 had the highest protein content (3.8 %), while the G11 strain cultivated in June 2013 had the lowest protein content (2.3 %). The species *Gelidium amansii* (red macroalgae) was recently reported to have a protein content of around 14.5 % [10, 29]. The insoluble aromatic content has not been documented in the literature and was quantified via hydrolysis with sulfuric acid [30]. However, *K. alvarezii* contains proteins; thus, the protein content of the acid hydrolysate was assayed. There was no significant difference between the protein content of the biomass (Table 1) and the corresponding acid hydrolysate (data not shown). All fractions retained on the filters that did not contain protein were considered as insoluble aromatics. The average insoluble aromatic content was 2.6 %. Galacturonic acid was detected in small amounts at an average of 1.2 %.

Hydroxymethylfurfural was also detected in the strains since this compound is derived from oxidation of glucose and galactose under acidic and high temperature conditions. However, the average value was 2.5 %, which is similar to that obtained from the hydrolysis process employing sulfuric acid and lignocellulosic biomass [44]. Only traces of furfural were detected in the samples (data not shown). The last component detected in the strains was lipid. The average lipid content was 0.6 %, similar to that documented for the same species (0.7 %) [24, 38]. The lipid content of the species *Gelidium amansii* (red macroalgae) was recently reported to be around 1 % [10, 29].

**Fig. 2:**
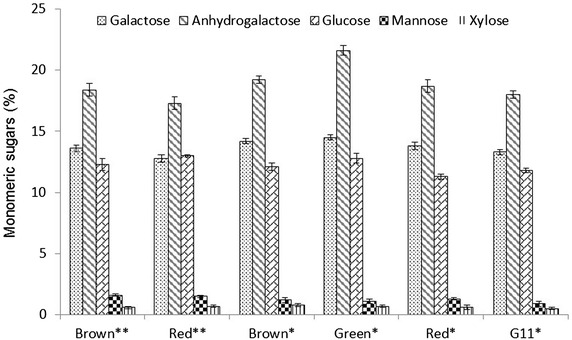
Sugar composition monomeric different strains of *K. alvarezii*. Contents present in percentage (g/100 g of original material in dry basis). *Asterisks* Cultivation for June 2013, *Two* *asterisks* cultivation for May 2013. All reported data are the average values followed by their standard deviations

**Fig. 3:**
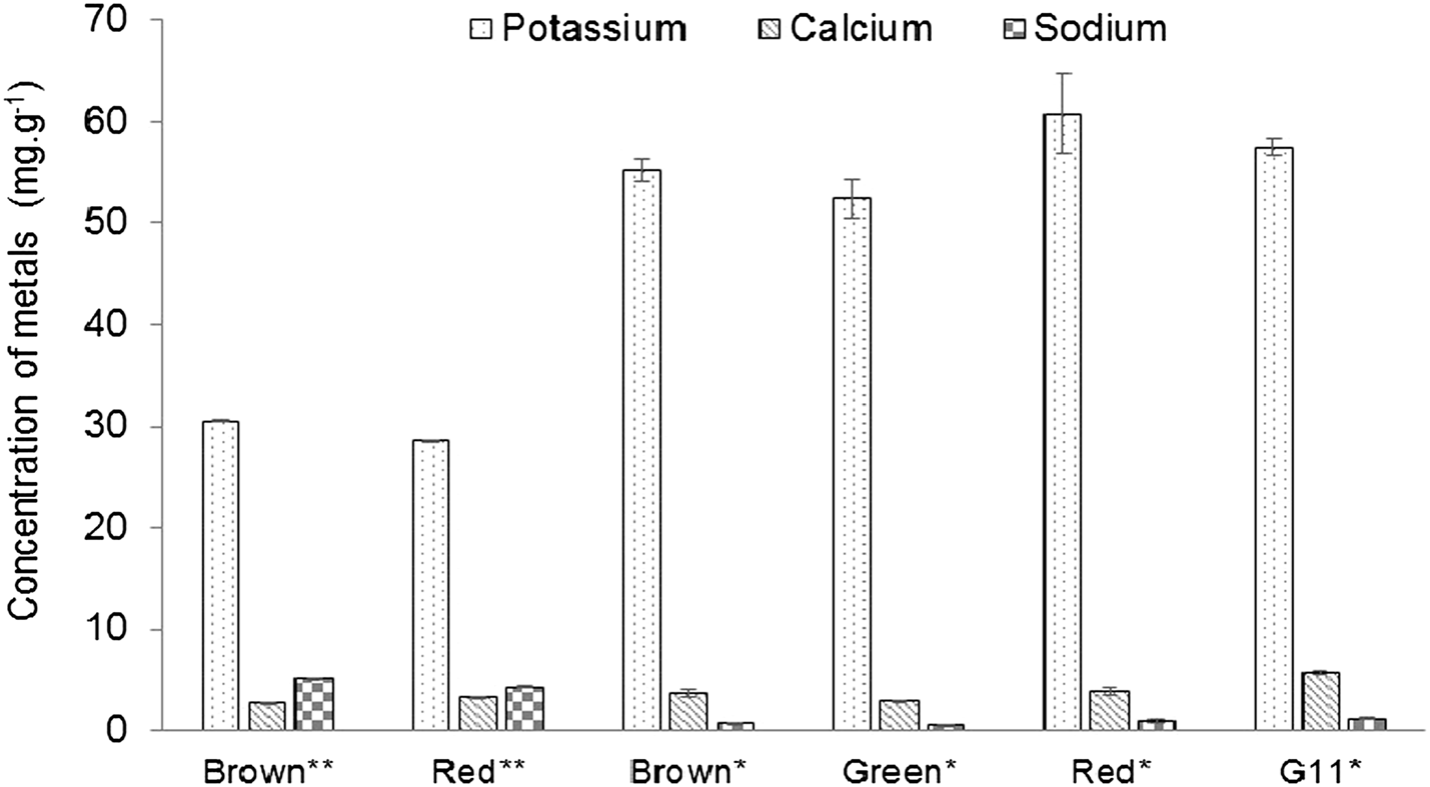
Composition of metals of different strains of *K. alvarezii*. Contents present in mass (milligrams/grams of original material in dry basis). *Asterisks* Cultivation for June 2013, *Two* *asterisks* cultivation for May 2013. All reported data are the average values followed by their standard deviations

In addition to the total carbohydrates, the profile of monomeric sugars in the *K. alvarezii* strains was also analyzed (Fig. 2). HPLC–MS analysis indicated the presence of anhydrogalactose, galactose, glucose, mannose, and xylose. In addition, rhamnose was detected in trace amounts due to the small portion of pectin in *K. alvarezii* biomass, derived from galacturonic acid [45] (Table 1). Arabinose and cellobiose were not detected in the samples. The percentage of galactose, anhydrogalactose, glucose, mannose, and xylose in the strains ranged from 13.8–14.5, 17.3–21.6, 11.3–13.0, 0.9–1.6, and 0.5–0.8 %, respectively (Fig. 2). Thus, the major polysaccharides found in *K. alvarezii* were galactans (from galactose and anhydrogalactose), followed by glucans (from glucose). The metal composition of the different strains under investigation was further analyzed by assay of the ash from *K. alvarezii* (Table 1; Fig. 3). The main metals detected were potassium, calcium, and sodium (Fig. 3). In addition to these metals, traces of manganese, iron, and silicon were detected. Notably, the concentrations of potassium, calcium, and sodium in the strains ranged from 28.6 to 60.8, 2.7–5.7, and 0.5–5.1 mg g^−1^, respectively (Fig. 3). The strains grown in June 2013 had higher potassium and lower sodium contents than those grown in May 2013 (Fig. 3).

### Carrageenan processing and obtaining residue from two selected strains

**Fig. 4:**
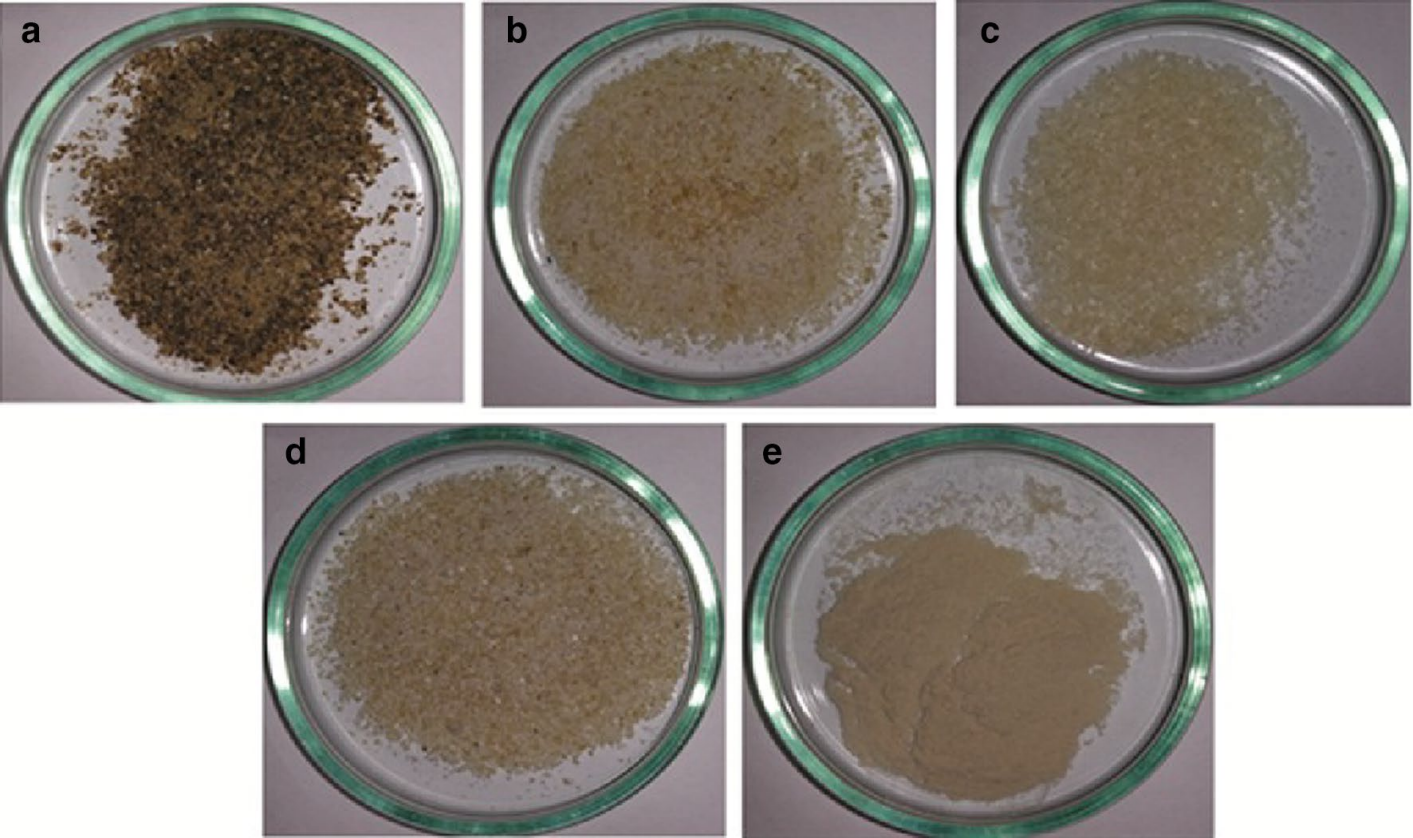
Residual solids of brown strain cultivation for May 2013 after treatment with 6 % KOH (w/v) and subsequently extraction of semi-refined carrageenan. **a** Untreated material, **b** treated with KOH 6 % (w/v) material, **c** semi-refined carrageenan, **d** residue and **e** commercial carrageenan

The brown and red strains grown in May 2013 were selected due to their higher productivity and growth rates. Another parameter that could have been used was the total carbohydrate content (Table 1), but these values did not vary greatly between the strains, thus the productivity and growth rates were taken into account. The yields of biomass from the brown and red strains treated with 6 % KOH solution (w/v) were 89.3 and 89.5 %, respectively (Table 2). The treated biomass became clearer compared to untreated biomass (Fig. 4a, b). Among all the components detected in the biomass (Table 1) from the four *K. alvarezii* strains, the following components were chosen for analysis of the chemical composition for the two selected strains: galactan, glucan, ash, proteins, insoluble aromatics, and sulfate groups (Table 2). The values of these components were similar for the untreated biomass and treated biomass, with the exception of the protein content (which was reduced by approximately 90 %) and the ash (which showed an accumulation of approximately 17 %) in the case of treated biomass. The dissolution of proteins is common in alkaline medium, while the accumulation of ash reflects the adsorption of potassium in the material upon treatment with 6 % KOH (w/v) [46] (Table 2).

**Table 2:** Yield and chemical composition of brown and red strains from *K. alvarezii* before and after treatment with 6 % (w/v) KOH and subsequently extracted with hot water (carrageenan and residue production)

Samples	Strain	Yield of sample (g/100 g of material) (%)	Galactan (%)	Glucan (%)	Ashes (%)	Proteins (%)	Insoluble aromatics (%)	Sulfate groups (%)
*Components of samples (% on pulp basic)*								
Untreated	Brown	100	33.0 ± 0.4	12.7 ± 0.5	14.6 ± 0.1	3.8 ± 0.5	2.4 ± 0.1	9.6 ± 0.2
Treated with KOH		89.3 ± 0.9	32.4 ± 0.8	13.5 ± 0.6	21.7 ± 0.1	0.5 ± 0.1	2.2 ± 0.2	10.4 ± 0.3
Residue		23.0 ± 0.5	7.2 ± 0.3	54.6 ± 0.4	14.9 ± 0.1	0.5 ± 0.1	3.9 ± 0.1	8.4 ± 0.1
Carrageenan		63.5 ± 0.6	42.6 ± 0.9	nd	24.2 ± 0.1	0.3 ± 0.1	1.1 ± 0.1	13.3 ± 0.2
Untreated	Red	100	31.3 ± 0.8	13.5 ± 0.1	16.0 ± 0.2	2.5 ± 0.3	1.5 ± 0.2	10.1 ± 0.3
Treated with KOH		89.5 ± 0.5	35.6 ± 1.2	14.1 ± 0.7	21.6 ± 0.1	0.4 ± 0.1	1.8 ± 0.2	10.6 ± 0.5
Residue		27.8 ± 1.2	9.7 ± 0.2	50.2 ± 1.2	10.1 ± 0.1	0.4 ± 0.1	3.2 ± 0.5	8.7 ± 0.1
Carrageenan		60.0 ± 1.5	46.6 ± 1.1	nd	28.5 ± 0.3	0.3 ± 0.1	1.4 ± 0.3	14.0 ± 0.3
Commercial carrageenan	–	–	32.5 ± 0.8	0.4 ± 0.1	34.8 ± 0.1	0.5 ± 0.2	0.7 ± 0.1	12.8 ± 0.1
*Components of samples (% of original material)*								
Untreated	Brown	100	33.0 ± 0.4	12.7 ± 0.5	14.6 ± 0.1	3.8 ± 0.5	2.4 ± 0.1	9.6 ± 0.2
Treated with KOH		89.3 ± 0.9	28.9 ± 0.8	12.0 ± 0.6	19.4 ± 0.1	0.4 ± 0.1	1.9 ± 0.2	9.3 ± 0.3
Residue		23.0 ± 0.5	1.7 ± 0.3	12.6 ± 0.4	3.4 ± 0.1	0.1 ± 0.1	0.9 ± 0.1	1.9 ± 0.1
Carrageenan		63.5 ± 0.6	27.0 ± 0.9	nd	15.4 ± 0.1	0.2 ± 0.1	0.7 ± 0.1	8.4 ± 0.2
Untreated	Red	100	31.3 ± 0.8	13.5 ± 0.1	16.6 ± 0.2	2.5 ± 0.3	1.5 ± 0.2	10.1 ± 0.3
Treated with KOH		89.5 ± 0.5	31.8 ± 1.2	12.6 ± 0.7	19.3 ± 0.1	0.4 ± 0.1	1.6 ± 0.2	9.5 ± 0.5
Residue		27.8 ± 1.2	2.7 ± 0.2	13.9 ± 1.2	2.8 ± 0.1	0.1 ± 0.1	0.9 ± 0.5	2.4 ± 0.1
Carrageenan		60.0 ± 1.5	28.0 ± 1.1	nd	17.1 ± 0.3	0.2 ± 0.1	0.8 ± 0.3	8.4 ± 0.3

Contents present in percentage (g/100 g of basic and original material in dry basis)

Cultivation for May 2013, commercial carrageenan = Sigma and nd = not detected. All reported data are the average values followed by their standard deviations. Pulp basic (data representing the biomass without considering a mass balance) and original material (data corrected considering the yield of the process, i.e., performing a mass balance)

The yields of extracted carrageenan and the residue obtained from the treated biomass were, respectively, 63.5 and 23 % for the brown strain and 60 and 27.8 % for the red strain (Table 2). The carrageenan was obtained as a translucent and soft material, while the residue was an opaque and hard material (Fig. 4c, d). The content of galactan, glucan, ash, protein, insoluble aromatics, and sulfate groups in the residues from the brown and red strains was 7.2 and 9.7 % (galactan), 54.6 and 50.2 % (glucan), 14.9 and 10.1 % (ash), 0.5 and 0.4 % (protein), 3.9 and 3.2 % (insoluble aromatics), and 8.4 and 8.7 % (sulfate groups), respectively (Table 2). The galactan content in the residue from the strains had lower and higher glucan contents than that of the samples treated with 6 % KOH (w/v) (Table 2). The galactan, ash, protein, insoluble aromatics, and sulfate groups contents of carrageenan from the brown and red strains were 42.6 and 46.6 % (galactan), 24.2 and 28.5 % (ash), 0.3 and 0.3 % (protein), 1.1 and 1.4 % (insoluble aromatics), and 13.3 and 14.0 % (sulfate groups), respectively (Table 2). The compositional profile of carrageenan obtained from both strains was similar to that of commercial carrageenan; the main components detected were galactan, ash, and sulfate groups (Table 2). For direct comparison of the chemical composition of the residue and the macroalgal material treated with 6 % KOH, a mass balance was required. The galactan, ash, protein, insoluble aromatics, and sulfate group contents of the residues from the brown and red strains were reduced by 94 and 91 % (galactan), 82 and 83 % (ash), 75 % for both (protein), 53 and 44 % (insoluble aromatics), and 80 and 75 % (sulfate groups), respectively. However, the glucan content in the residue was not reduced for either strain (Table 2). After completing the process of obtaining carrageenan (treatment with 6 % KOH solution and subsequent extraction with hot water), the yields of carrageenan and residue were, respectively, 56.7 and 20.5 % for the brown strain and 53.6 and 24.8 % for the red strain. The overall yields of semi-refined carrageenan were similar to those reported in the literature [27, 32].

The metal content of the samples treated with 6 % KOH solution (w/v) and extracted with hot water was also assessed. The three main metals evaluated were potassium, calcium, and sodium (Table 3). The chemical composition of the original material treated with 6 % KOH solution (w/v) for both strains indicated accumulation of potassium (approximately 20 %) and reduction of the sodium content (by approximately 73 %). The calcium content was similar to that of the untreated samples (Table 3). The potassium, calcium, and sodium contents of the residues from the brown and red strains were 7.2 and 9.7 % (potassium), 54.6 and 50.2 % (calcium), and 14.9 and 10.1 % (sodium), respectively. Assessment of the metal content of the carrageenan extracted from the brown and red strains revealed lower levels of metals compared to that found in the residue. Specifically, the potassium, calcium, and sodium contents of the carrageenan from the brown and red strains were 0.5 and 0.4 % (potassium), 3.9 and 3.2 % (calcium), and 8.4 and 8.7 % (sodium), respectively (Table 3). For direct comparison of the chemical composition of the residue and the macroalgal material treated with 6 % KOH, a mass balance was required. From the mass balance, the metal contents of the residues from the brown and red strains were, respectively, reduced as follows: potassium (86.6 and 84.7 %), calcium (70.0 and 45.8 %), and sodium (61.5 and 58.3 %) (Table 3).

**Table 3:** Yield and metals composition of brown and red strains from *K. alvarezii* before and after treatment with 6 % (w/v) KOH and subsequently extracted with hot water (carrageenan and residue production)

Samples	Strains	Yield of sample (g/100 g of material) (%)	Potassium (mg g^−1^)	Calcium (mg g^−1^)	Sodium (mg g^−1^)
*Components of samples (mg* *g* ^*−1*^ *on pulp basic)*					
Untreated	Brown	100	30.5 ± 0.01	2.7 ± 0.01	5.1 ± 0.01
Treated with KOH		89.3 ± 0.9	42.7 ± 0.01	3.4 ± 0.01	1.5 ± 0.08
Residue		23.0 ± 0.5	22.3 ± 0.07	3.8 ± 0.06	2.3 ± 0.04
Carrageenan		63.5 ± 0.6	53.5 ± 0.01	2.7 ± 0.01	1.2 ± 0.01
Untreated	Red	100	28.6 ± 0.01	3.3 ± 0.02	4.3 ± 0.05
Treated with KOH		89.5 ± 0.5	36.5 ± 0.02	2.7 ± 0.01	1.3 ± 0.04
Residue		27.8 ± 1.2	18.2 ± 0.04	4.0 ± 0.01	2.0 ± 0.06
Carrageenan		60.0 ± 1.5	48.6 ± 0.02	2.6 ± 0.01	1.2 ± 0.01
Commercial carrageenan	–	–	72.8 ± 0.3	25.8 ± 0.2	8.6 ± 0.1
*Components of samples (mg* *g* ^*−1*^ *of original material)*					
Untreated	Brown	100	30.5 ± 0.01	2.7 ± 0.01	5.1 ± 0.01
Treated with KOH		89.3 ± 0.9	38.1 ± 0.01	3.0 ± 0.01	1.3 ± 0.08
Residue		23.0 ± 0.5	5.1 ± 0.07	0.9 ± 0.06	0.5 ± 0.04
Carrageenan		63.5 ± 0.6	34.0 ± 0.01	1.7 ± 0.01	0.8 ± 0.01
Untreated	Red	100	28.6 ± 0.01	3.3 ± 0.02	4.3 ± 0.05
Treated with KOH		89.5 ± 0.5	32.7 ± 0.02	2.4 ± 0.01	1.2 ± 0.04
Residue		27.8 ± 1.2	5.0 ± 0.04	1.1 ± 0.01	0.5 ± 0.06
Carrageenan		60.0 ± 1.5	29.1 ± 0.02	1.5 ± 0.01	0.7 ± 0.01

Contents present in mass (milligrams/grams of basic and original material in dry basis)

Cultivation for May 2013 and commercial carrageenan = Sigma. All reported data are the average values followed by their standard deviations. Pulp basic (data representing the biomass without considering a mass balance) and original material (data corrected considering the yield of the process, i.e., performing a mass balance)

### Enzymatic hydrolysis of fractions from *K. alvarezii*

Direct enzymatic hydrolysis of the untreated and treated (with 6 % KOH) materials and the residue can provide an indication of their digestibility and the suitability for macroalgae bioethanol production processes [25, 26, 30]. Such processes are also applied to extraction of carrageenan from *K. alvarezii*. Therefore, a mixture of commercial cellulases was used herein to hydrolyze the fractions to obtain monomeric sugars.

**Fig. 5:**
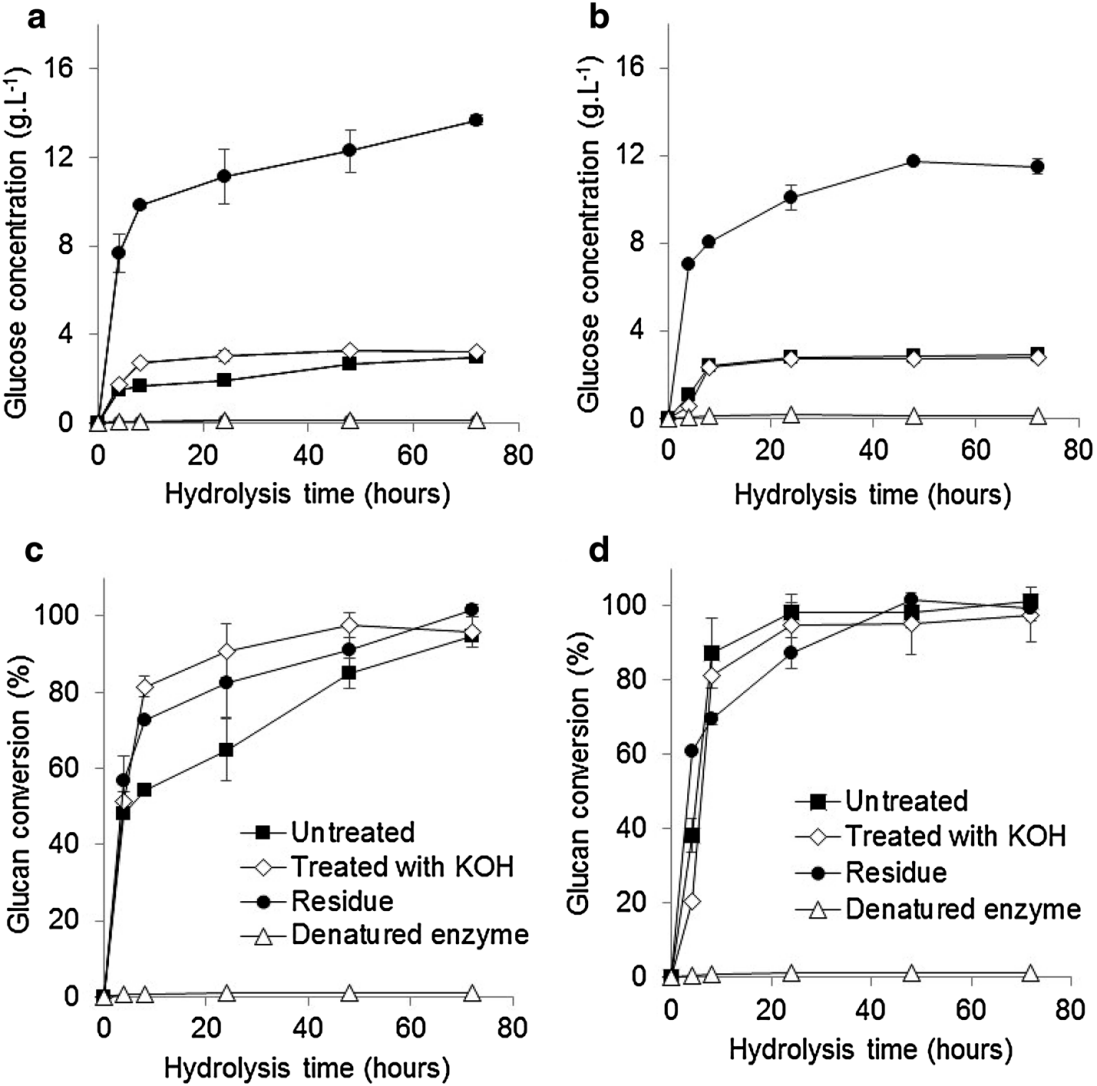
Glucose concentration and glucan conversion over time for enzymatic hydrolysis from *K. alvarezii* fractions cultivation for May 2013. **a** Glucose concentration of brown strain. **b** Glucose concentration of red strain. **c** Glucan conversion of brown strain. **d** Glucan conversion of red strain. (*square filled*) untreated material, (*lozenge open*) treated with KOH 6 % (w/v) material, (*ball filled*) residue and (*triangle open*) residue plus enzyme denature. All *symbols* apply to the graphs **a**–**d**. All reported data are the average values followed by their standard deviations

The glucose concentration in the untreated and treated materials and the residue from the brown and red strains after a 72 h enzymatic hydrolysis period were 3.2 and 2.8 g L^−1^ (untreated), 3.2 and 2.8 g L^−1^ (treated), and 13.7 and 11.5 g L^−1^ (residue), respectively (Fig. 5a, b). In most cases, the glucan conversion after 72 h of enzymatic hydrolysis was found to be 100 % (Fig. 5c, d). Enzymatic hydrolysis of the residues produced the highest concentrations of glucose given that the residues were rich in glucan (Table 2). The maximum rate of hydrolysis of the residues was 1.8 g L^−1^ h^−1^, whereas the hydrolysis rates of the untreated and treated samples were 0.3 g L^−1^ h^−1^. The high rate of hydrolysis of the residues is associated with its greater capacity to adsorb cellulases due to the glucan-rich nature of the residues (Fig. 5a, b; Table 2). The commercial enzyme extract could hydrolyze the glucans present in all fractions studied without any recalcitrance of the material (Fig. 5c, d). Galactase was not present in the commercial enzymes used for hydrolysis, and the untreated and treated fractions remained rich in galactans (Tables 1, 2). Since galactase activity was not detected for the enzymes used, the polysaccharide fraction comprising galactans was not hydrolyzed.

## Conclusions

In summary, the current evaluation of four different *K. alvarezii* strains demonstrated differential rates of productivity and growth; nevertheless, all strains had comparable total carbohydrate levels, the main component found in *K. alvarezii* biomass. The main carbohydrate polymers were galactan and glucan. Other important components were ash (mainly comprised calcium, potassium, and sodium) and sulfate groups.

Semi-refined carrageenan and its residue were successfully obtained from the two selected strains. Upon enzymatic hydrolysis, the residue yielded high concentrations of glucose, with complete conversion of glucan. These results highlight the viability of this byproduct of carrageenan extraction as a monomeric sugar for the eventual production of bioethanol. Since this residue is considered waste and not a food source, this method would be a fourth generation model for the production of biofuels. In summary, we have demonstrated a novel aspect to the biorefining of *K. alvarezii* for developing not only carrageenan, but also the bioproduct glucose. Such insights can further advance this field towards better design of biofuel production strategies.

## Abbreviations

FPU: filter paper unit
HPLC/MS: liquid chromatography/mass spectrophotometry
HPLC: high-performance liquid chromatography
UV: ultraviolet
ESI: electrospray ionization
GHG: greenhouse gas
GW: global warming
SP: São Paulo
SD: standard deviation
